# Cassava mosaic disease and its management in Southeast Asia

**DOI:** 10.1007/s11103-021-01168-2

**Published:** 2021-07-09

**Authors:** Ayaka Uke, Hiroki Tokunaga, Yoshinori Utsumi, Nguyen Anh Vu, Pham Thi Nhan, Pao Srean, Nguyen Huu Hy, Le Huy Ham, Luis Augusto Becerra Lopez-Lavalle, Manabu Ishitani, Nguyen Hung, Le Ngoc Tuan, Nguyen Van Hong, Ngo Quang Huy, Trinh Xuan Hoat, Keiji Takasu, Motoaki Seki, Masashi Ugaki

**Affiliations:** 1 Graduate School of Frontier Sciences, The University of Tokyo, Kashiwa, Chiba Japan; 2 Center for Sustainable Resource Science, RIKEN, Yokohama, Kanagawa Japan; 3 International Laboratory for Cassava Molecular Breeding (ILCMB), AGI, Hanoi, Vietnam; 4 National Key Laboratory for Plant Cell Technology, Agricultural Genetics Institute (AGI), Hanoi, Vietnam; 5 Hung Loc Agricultural Research Center (HLARC), Dong Nai, Vietnam; 6 University of Battambang (UBB), Battambang, Cambodia; 7 International Center for Tropical Agriculture (CIAT), Cali, Colombia; 8 Sub-Department of Plantation and Plant Protection of Tay Ninh Province, Hanoi, Vietnam; 9 Plant Protection Research Institute (PPRI), Hanoi, Vietnam; 10 Faculty of Agriculture, Kyushu University, Fukuoka, Japan; 11 RIKEN Cluster for Pioneering Research, Saitama, Japan; 12 Kihara Institute for Biological Research, Yokohama City University, Yokohama, Kanagawa Japan

**Keywords:** *Manihot esculenta*, Cassava mosaic begomoviruses, Seed system, Plant diseases, LAMP

## Abstract

**Key message:**

Status of the current outbreak of cassava mosaic disease (CMD) in Southeast Asia was reviewed. Healthy cassava seed production and dissemination systems have been established in Vietnam and Cambodia, along with integrated disease and pest management systems, to combat the outbreak.

**Abstract:**

Cassava (*Manihot esculenta* Crantz) is one of the most important edible crops in tropical and subtropical regions. Recently, invasive insect pests and diseases have resulted in serious losses to cassava in Southeast Asia. In this review we discuss the current outbreak of cassava mosaic disease (CMD) caused by the Sri Lankan cassava mosaic virus (SLCMV) in Southeast Asia, and summarize similarities between SLCMV and other cassava mosaic begomoviruses. A SATREPS (Science and Technology Research Partnership for Sustainable Development) project “Development and dissemination of sustainable production systems based on invasive pest management of cassava in Vietnam, Cambodia and Thailand”, was launched in 2016, which has been funded by The Japan International Cooperation Agency (JICA) and The Japan Science and Technology Agency (JST), Japan. The objectives of SATREPS were to establish healthy seed production and dissemination systems for cassava in south Vietnam and Cambodia, and to develop management systems for plant diseases and insect pests of cassava. To achieve these goals, model systems of healthy seed production in Vietnam and Cambodia have been developed incorporating CMD-resistant planting materials through international networks with The International Center for Tropical Agriculture (CIAT) and The International Institute of Tropical Agriculture (IITA).

**Supplementary Information:**

The online version contains supplementary material available at 10.1007/s11103-021-01168-2.

## Introduction

Cassava (*Manihot esculenta* Crantz, family Euphorbiaceae) is one of the most important edible crops in the world. Although Africa currently has the largest production area in the world, cultivation as a food crop and as a source for biofuel has been increasing in Southeast Asia, where total cassava production is now more than 56 Mt/year (Fauquet et al. 2018; Marx 2019). Cassava production is affected by dozens of factors across the producing countries, including diseases (Tokunaga et al. 2018). In particular, two viral diseases, cassava mosaic disease (CMD) and cassava brown streak disease (CBSD), present the most serious threats to global production. To date no reports of CBSD exist outside Africa. CMD was first reported in Southeast Asia at Ratanakiri, Cambodia in 2015, with its causal agent subsequently identified as Sri Lankan cassava mosaic virus (SLCMV) (Wang et al. 2016). Since that time the virus has been spreading rapidly (Minato 2019). Planting resistant varieties has been shown to be the most effective strategy for controlling the disease. However, CMD is new to Southeast Asia and appropriate resistant varieties have yet to be developed. A strategy for the management of the disease and the creation of a supply of healthy planting materials in farmer-preferred varieties represents an urgent need in Southeast Asia.

The SATREPS (Science and Technology Research Partnership for Sustainable Development) project “Development and dissemination of sustainable production systems based on invasive pest management of cassava in Vietnam, Cambodia and Thailand”, was launched in 2016 funded by the Japanese Government (Tokunaga et al. 2018). SATREPS objectives were to develop pest management technologies and a system for the production and cultivation of healthy seedlings. Notably, this project worked to construct a sustainable production system based on utilization of healthy planting materials by developing a market-based “triple-win” dissemination model that benefits the private sector, farmers, and the government. The project is a collaborative research program between the Agricultural Genetics Institute (AGI), Plant Protection Research Institute (PPRI), Hung Loc Agricultural Research Center (HLARC), Nong Lam University (NLU), Vietnam; University of Battambang (UBB), Cambodia; and the Rayong Field Crops Research Center (RFCRC), Thailand.

This review summarizes the main aspects of CMD in Southeast Asia, studies on management and control measures that have been, or could be, adopted in Southeast Asia, and opportunities for the use of these approaches on a large scale. SATREPS activities against CMD in Southeast Asia are also described.

## Outbreaks of SLCMV in Southeast Asia

**Fig. 1:**
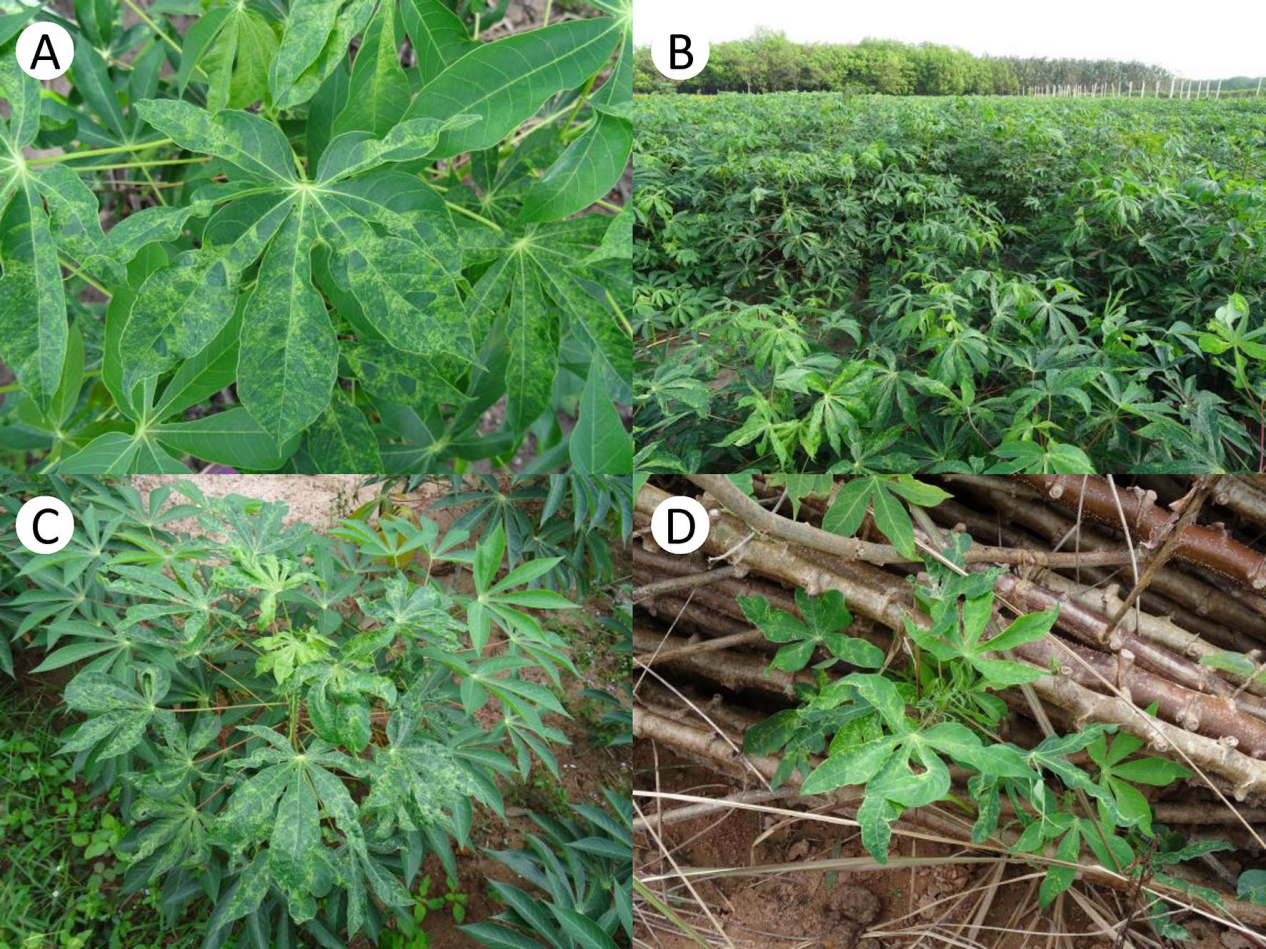
Cassava mosaic disease symptoms on cassava plants in Cambodia and Vietnam. **A** Mosaic symptoms on leaves. **B** Heavily CMD infected field. **C** Diseased plant. **D** Diseased plantlet sprouting from a seemingly healthy, yet diseased, stem

Cassava mosaic disease (CMD) is caused by cassava mosaic begomoviruses (CMBs) that belong to the genus *Begomovirus*, family *Geminiviridae*, and is a major constraint on cassava production in Africa and Asia. The genome of CMBs is bipartite, composed of two circular single-strands of DNA (DNA-A and -B), each approximately 2.8 kb in size. The strands are encapsulated in twinned icosahedral particles that replicate via the rolling-circle mechanism with double-stranded DNA intermediates. CMD symptoms can be clearly recognized and include stunted and distorted shoots and leaves with an evident pale green to yellow mosaic pattern (Fig. 1). Compromised leaf development and photosynthetic capacity causes reduced storage root yields. Planting of infected cuttings by farmers results in increasing yield losses over subsequent cropping cycles. There are 10 species of CMBs known in Africa and Asia, including African cassava mosaic virus (ACMV), cassava mosaic Madagascar virus (CMMGV), East African cassava mosaic virus (EACMV), East African cassava mosaic Cameroon virus (EACMV), East African cassava mosaic Kenya virus (EACMKV), East African cassava mosaic Malawi virus (EACMMV), East African cassava mosaic Zanzibar virus (EACMZV), South African cassava mosaic virus (SACMV) (Zerbini 2017), Indian cassava mosaic virus (ICMV) and SLCMV (Saunders et al. 2002).

Production of cassava on the Indian subcontinent has been severely affected by CMD which is caused by ICMV and SLCMV. The genome sequences of ICMV and SLCMV were first reported by Hong et al. (1993) and Saunders et al. (2002), respectively. The DNA-A and DNA-B sequences of ICMV are 65% and 30% identical to those of ACMV. Although SLCMV shares Rep-binding iteron sequences with ACMV, its whole genome sequence exhibits greater identity to the genome of ICMV (Patil and Fauquet 2009). While CMD has been present in Africa since the 19th century it was not known in Southeast Asia until May 2015, when the first CMD outbreak was observed in Cambodia. While the causal agent was identified as SLCMV (Wang et al. 2016), the first location of virus introduction and its method of dissemination remain unclear. The disease area has been expanding not only within Cambodia but had spread into Vietnam and China by 2017, and reached Thailand in 2019 (Uke et al. 2018; Minato et al. 2019; Wang et al. 2019; Leiva et al. 2020; Siriwan et al. 2020). After the discovery of CMD in Southeast Asia a preliminary survey was performed to determine the effect of SLCMV on cassava root yields in Vietnam. We found that cassava root yield and starch content declined 16–33% and 22–38%, respectively, when SLCMV-infected cuttings were used at planting time, compared to the use of healthy disease-free cuttings (Suppl. Table 1). These findings are consistent with those in Africa where a reduction in tuberous root weight and total yield associated with CMD was also described (Fauquet and Fargette 1990; Owor et al. 2004).

The first reported Vietnamese isolate of SLCMV (DNA-A, GenBank no.: LC312131; DNA-B, LC312130) was found to possess sequences highly homologous with Cambodian isolates (DNA-A, KT861468; DNA-B, KT861469); sharing 99.8% identity (Uke et al. 2018). An additional 16 isolates were collected in Cambodia and Vietnam with molecular analysis in progress. Several serological and molecular methods are available for diagnosing SLCMV. Serological methods are most appropriate for mass screening while molecular methods, including PCR, are highly sensitive and can be used for detection and sequencing to the strain level (Tokunaga et al. 2018). Most of these methods, however, require substantial amounts of labor and time and specialized laboratory equipment, and are not suitable for on-site detection in the field. Loop-mediated isothermal amplification (LAMP) is a highly sensitive and rapid molecular method and is now available for on-site detection of SLCMV in diseased leaves and stems.

## Manner of transmission

CMBs are naturally transmitted by a group of whiteflies, the *Bemisia tabaci* species complex, which is composed of multiple cryptic species, in a non-propagative and circulative manner (Islam et al. 2018). The species status of *B. tabaci* has been determined by phylogenetic relationship of nucleotide sequences of the mitochondrial cytochrome c oxidase subunit I gene (*cox1*). At least 24 species have been identified (De Barro et al. 2011). Whiteflies collected from diseased cassava leaves in Cambodia and Vietnam were sequenced. All were classified as *B. tabaci* Asia II 1 (Wang et al. 2016; Uke et al. 2018). Inoculation experiments conducted using whiteflies collected in Vietnam demonstrated that the virus can be transmitted from SLCMV infected cassava to healthy plants (Tran Van Chien, personal communication). Since cassava is a vegetatively propagated crop, cassava stems that infected with CMBs can also serve as a source of disease. While whiteflies play a role as disease vectors in short distance dissemination, within 20 m (Maruthi et al. 2017), distribution networks of cassava planting materials, especially via diseased stem cuttings (Delaquis 2018), play a significant role in long distance dissemination. Notably, healthy and diseased stems with the leaves removed, are morphologically indistinguishable (Fig. 1D); making the commercial distribution of CMD diseased vegetative planting materials a major concern for rapid spread of CMD across Southeast Asia. Use of insecticides to reduce whitefly density could limit short-distance dissemination; however, over use of insecticides could accelerate the displacement of existing *B. tabaci* species to the *B. tabaci* Mediterranean (biotype Q) that are resistant to insecticides currently used to control this pest (Horowitz et al. 2014). Development of an integrated pest management (IPM) is therefore highly recommended to control whiteflies in the field and to prevent the development of insecticide-resistant whiteflies that could spread into other regions that cultivate cassava.

## SATREPS project

**Fig. 2:**
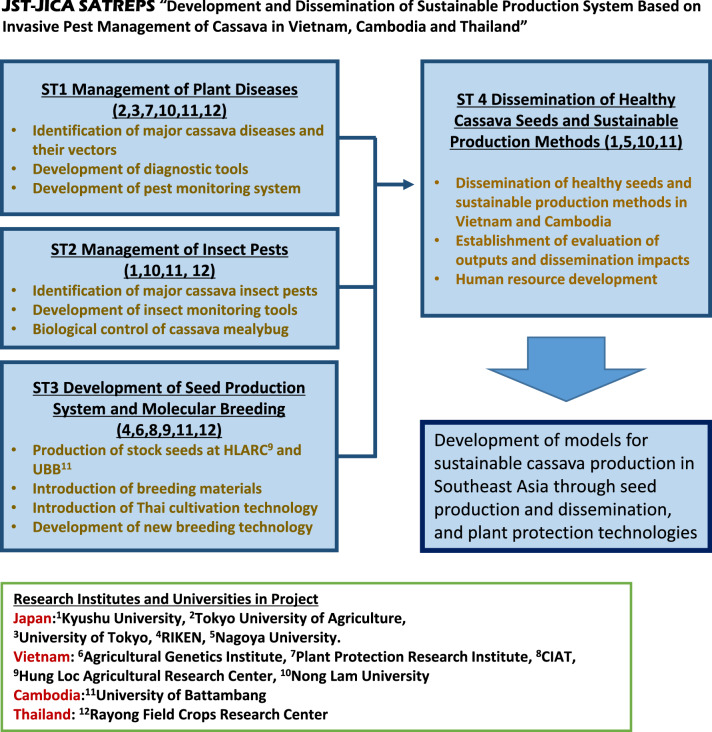
SATREPS project outline

Cassava production in Southeast Asia did not suffer from serious insect pests and diseases until the early 2000s. Since then cassava mealybug *Phenacoccus manihoti* Matile‐Ferrero (Hemiptera: *Pseudococcidae*) and the phytoplasma disease, cassava witches’ broom (Graziosi 2016) have begun to cause serious damage to cassava in the region. The SATREPS project was initiated to address these growing pest problems, with the major objective to develop sustainable cassava production systems in Southeast Asia. Various research institutes and universities in Vietnam, Cambodia, Thailand and Japan joined the project (Fig. 2). The project consists of four sub-teams covering plant pathology, entomology, seed production and molecular breeding, plus extension and socioeconomic analysis (Fig. 2). The project is designed to establish healthy cassava seed production and dissemination systems in south Vietnam and Cambodia, and to develop management systems for cassava diseases and insect pests. Model systems of healthy seed production and dissemination in Vietnam and Cambodia are being implemented to address the first objective. Cassava stock seed production systems have been established at HLARC, Dong Nai, Vietnam and at UBB, Battambang, Cambodia. Conservation and propagation of healthy seed stocks of major cassava varieties have been established at HLARC and UBB using stock seed production fields and screenhouses, and tissue culture and aeroponics in laboratory facilities (Tokunaga et al. 2018, 2019). Several local farmers in Dong Nai, Vietnam, and Battambang and Banteay Meanchey, Cambodia have been engaged to produce healthy cassava seeds using the stock cassava seeds produced at HLARC and UBB. Because no CMD resistant varieties are available it is critical for such seed producers to monitor for CMD among other plant diseases and insect pests. Local farmers are being trained to use methods as record sheets and a field guide to identify insect pests and their natural enemies on cassava and to monitor for plant diseases and insect pests in the area.

Major plant diseases and insect pests of cassava in Vietnam and Cambodia have been identified in order to develop appropriate pest management systems (Tokunaga et al. 2018; Uke et al. 2018). When the project launched, the main disease target was cassava witches’ broom caused by a phytoplasma. However, with the rapid expansion and concerns around CMD, SATREPS made adjustments to address this growing problem. Field surveys were conducted in Vietnam and Cambodia and protocols developed, including the design of CMD-specific primers to allow high volume disease screening. LAMP primers and protocols have also been developed for on-site disease detection and diagnosis and with LAMP detection kits employed for identification of CMD and cassava witches' broom. Notably, an image diagnosis system for major plant diseases and insect pests of importance in Southeast Asia is being developed and will be implemented through a social network service to help farmers and extension workers identify plant diseases and insect pests (Uke et al. 2019).

## Development of a healthy seedling production and monitoring system

**Fig. 3:**
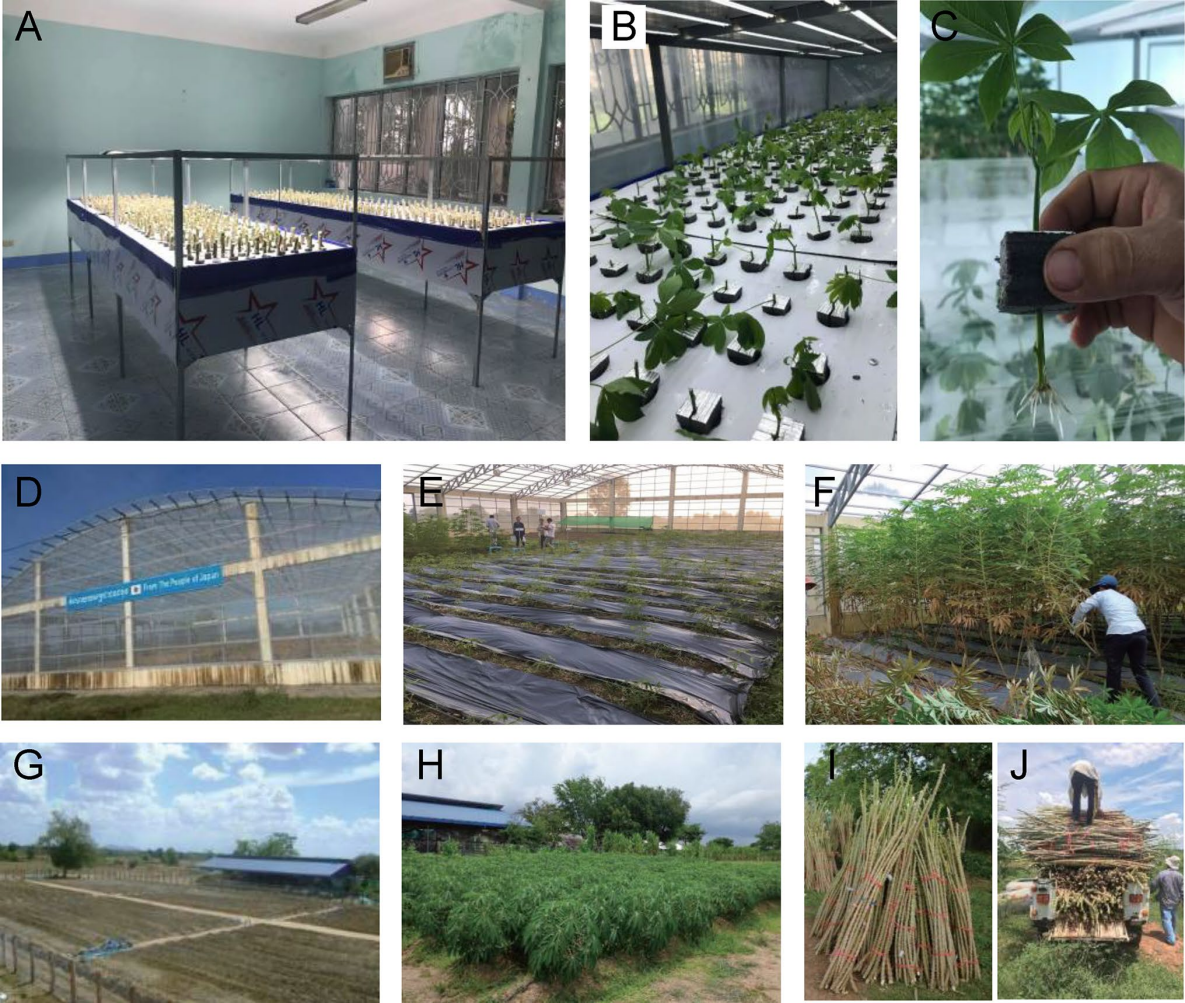
Facilities in the seed system at HLARC and UBB. **A**–**C** Aeroponic culture. **A** Aeroponic equipment in a growth room at HLARC. **B** Multiplication cycle using newly obtained shoots. **C** Plant phenotype after cultivation for 7 days. **D**–**H** Screenhouse cultivation. **D** Screenhouse at UBB cassava center. **E** Maintenance of the initial stock of plant materials in a vector-free facility. **F** Harvesting of plant materials. **G**–**H** Stock seed production in the field. **G** Overview of the stock seed production field at the UBB cassava center. **H** Plant growth. **I** Stock seeds. **J** Transport to a healthy seed production field

Efficient methods for cassava propagation and a system for production of healthy planting material have been established in HLRAC and UBB as part of the SATREPS project (Fig. 3). Cassava is typically multiplied by planting lignified-stem cuttings directly in the field (Howeler and Maung Aye 2014). The multiplication rate using this system is low at approximately ten times per year (FAO 2013; Howeler and Maung Aye 2014). Long term cultivation under field conditions increases the risk of disease infection. Therefore, the utilization of more efficient protocols is required at seed production centers.

Plant tissue culture is an option for the establishment of a disease-free system (Mafla et al. 2010) and provides three main advantages. Firstly, tissue culture plants can be quickly propagated using an optimal growth medium; thereby providing a fast-rate multiplication opportunity. Secondly, pathogens can be eliminated if a meristem culture approach is utilized and thirdly, because tissue culture derived plants are propagated in vitro*,* the risk of disease infection is eliminated at this stage. Large scale propagation by tissue culture, however, requires a high initial monetary investment to establish and support facilities and equipment, and to sustain trained technical staff. Tissue culture-derived plantlets also require up 10–12 weeks for soil acclimation and growth to reach a height of 25 cm, an adequate height for field planting (Tokunaga et al. 2018, 2019). While tissue-culture platforms would not be required in multiple locations in each country, it is considered that tissue culture facilities should therefore not serve as the principal propagation source for disease-free cassava plants, but can act as germplasm stocks for certified disease and pest free elite varieties.

As an alternative we recently developed an aeroponic culture system that can quickly generate large numbers of plants (Tokunaga et al. 2019). Notably, young stems (4–6 weeks old) can be used as planting material for this system. Plant propagation can also be repeated period every 3–6 weeks, allowing rapid increases in the number of propagated plants through continuous iterations of the system. Using this system, we can readily produce 1000 seedlings every 4–6 weeks. The advantages of the aeroponic system of propagation over the tissue culture and traditional methods is that any stage/kind of cassava plant can be used as a starting source; including tissue-culture-derived plants, young seedlings 4–6 weeks old, and even lignified stems up to 8–9 months old.

A further propagation method is the mini-cutting technique that employs small stem cuttings of approximately 5 cm in length that are planted in soil. The multiplication rate with this method is between 60 and 100 times per year (NurulNahar and Tan 2012). The technique has an advantage in cost over the aeroponic system and does not require hydroponic solutions and equipment. Since explants from relatively young plants (4–6 weeks) cannot be used as a propagation source in this method, the multiplication ratio is lower than in the aeroponic system. It is considered that the most appropriate and optimal method of propagation will vary for each country, propagation center, or farmer because each has a different level of technology and capacity.

The original plant materials used in the seed systems described here represent collections of new superior varieties or farmer-preferred varieties that have been confirmed to be CMD-free. Plants are maintained in vector-free conditions in a growth room or a screenhouse covered with a screen net to block admittance of whiteflies (Matsuura et al. 2005). These plants are used as the starting materials for propagation by the aeroponic mini-cutting techniques. Large-scale aeroponic equipment has been established in a growth room at HLARC. Efficient use of the rapid propagation systems alone is not sufficient to supply the large number of clean seedlings needed to meet demand in Vietnam. Therefore, propagation in the field must take place as the last step to produce large numbers of high-quality stems for deployment to farmers (Alves 2002). It is essential that producers at this stage regularly monitor disease symptoms during field-cultivation. Any CMD-symptomatic plants must be immediately removed; otherwise the proportion of CMD infected plants may increase even if CMD-free materials were initially provided as stock seedlings.

## Potential sources of CMD resistance

Identification of resistance sources and breeding for resistance have been a major focus in the battle against CMD (Malik 2020). To date, three sources of resistance, named CMD1, CMD2, and CMD3 have been identified, partially characterized, and used to develop CMD resistant cassava varieties. CMD1 is a recessive polygenic locus (Fregene et al. 2001) first reported in a wild relative of cassava, *Manihot glaziovii* Mull. Arg. (Ceara rubber) (Nichols 1947). CMD1 was introgressed into *M. esculenta* through interspecific-crossing and backcrossing, resulting in the release of over 200 improved IITA varieties in Africa (Manyong et al. 2000). CMD2, which is conferred by a dominant single locus, was identified in a tropical *M. esculenta* (TME) population in Africa and cultivated as landraces by farmers under wide-spread and prolonged CMD pressure (Akano et al. 2002). Varieties carrying CMD2 exhibit a high level of resistance to almost all CMBs and have the main source for CMD resistance breeding programs in Africa, Latin America, and Asia. CMD3 was initially produced by crossing TME and TMS genotypes (Lokko et al. 2005). Genetic analysis of a cultivar TMS 97/2205 and its offspring, however, revealed that CMD3 is a new quantitative trait locus (QTL) that provides putative resistance to CMD, which is not linked to CMD2 (Okogbenin 2012). The recent emergence of CMD in Cambodia, Vietnam, and Thailand led to the development and introduction of CMD-resistant materials by The International Center for Tropical Agriculture (CIAT) and The International Institute of Tropical Agriculture (IITA) into Thailand and Vietnam. C-33, a breeding line which contains CMD2 from CIAT, was introduced in the SATREPS project, among other lines, to provide a potential source of resistance to SLCMV. C-33 exhibited normal growth and remained asymptomatic in a high-SLCMV-pressure field in Tay Ninh province, Vietnam, where Asian cultivars exhibited CMD symptoms three months after planting. Grafting experiments also demonstrated that SLCMV was not transmitted from an infected rootstock to C-33 plants (Vu et al. 2020). These results convincingly suggest that CMD2 could be a strong source of resistance for breeding new varieties suitable for farmers in Southeast Asia (Supplementary Fig. 1).

## Molecular breeding

An important strategy that addresses the problem of CMD is to breed SLCMV-resistant cassava varieties using molecular breeding methods, such as marker-assisted selection and genome engineering. These approaches would facilitate the transfer of SLCMV resistance into superior cultivars in each region that are acceptable to local farmers and starch factories. In order to advance molecular breeding efforts in Vietnam, the International Laboratory for Cassava Molecular Breeding (ILCMB) was established in Vietnam in 2012 by partnerships between the Agricultural Genetics Institute (AGI), the Vietnamese Academy of Agricultural Sciences (VAAS) in Hanoi, Vietnam and CIAT (Malik et al. 2020). RIKEN also joined ILCMB as a core group in 2012 and the SATREPS project has supported their activity since 2016 (Utsumi et al. 2015; Tokunaga et al. 2018). The goal of ILCMB is to provide genetic solutions to the problems that cassava producers face in Southeast Asia. ILCMB operates as an open laboratory, and anyone can join as a member to contribute to cassava breeding research.

The most practical approach would be to incorporate marker selection into existing breeding programs in Southeast Asia. For example, DNA markers could be used to facilitate the rapid evaluation of CMD2-type resistance in breeding populations. However, the markers presently available are not tightly linked to the resistance locus. Recombination events occur between the resistance trait and the markers RME1 and NS158 at a level of 4% (4 cM) and 7% (7 cM), respectively (Carmo et al. 2015). Recently, de novo assemblies of the genomes of the African landraces TME3 (CMD2-type resistant) and 60,444 (CMD susceptible) have become available, and indicate that CMD2-associated markers align to an approximate 5 Mb region in chromosome 12 (Kuon 2019). A new high resolution map of the CMD2 locus would facilitate gene mapping studies designed to narrow this large region to identify CMD-resistance gene(s) at that locus. Breeding brings additional challenges, however. CMD2 was selected by West African farmers and exists in germplasm suited to the African environment and African consumers. Due to the heterozygotic nature of cassava, crossing African germplasm with Asian varieties will diminish the achievements of breeding programs carried out in Southeast Asia over the past several decades (Malik et al. 2020). Every time a cross is made, a large population of progeny must be recovered and selected to meet the required combination of superior agricultural traits required by Asian farmers, processors and consumers.

The long breeding-cycle of cassava is a major limiting factor for developing and implementing breeding programs. Research on the physiological mechanisms regulating flowering in cassava in the field and laboratory is being conducted to develop a cassava breeding strategy with a shortened breeding-cycle (Tokunaga et al. 2020). Flowering time in cassava varies and is dependent on the genotype and the environment (Keating et al. 1982; AZIZ 1984; Ceballos et al. 2004). The ability to regulate flowering and understand the molecular mechanisms controlling flowering in cassava would help to shorten the time needed to conduct cassava improvement programs. In the SATREPS project, we have attempted to develop floral induction technology. It is generally known that *FLOWERING LOCUS T* (*FT*) gene in *Arabidopsis* and orthologs in cassava and other species encode florigen, which is generated in leaves and transported via phloem to the shoot apex, where it promotes flowering (Corbesier 2007; Tamaki et al. 2007; Adeyemo et al. 2017; Bull et al. 2017; Odipio et al. 2020). We have examined floral regulation by grafting *FT*-over-expressing cassava with cultivars which are required for floral induction (Tokunaga et al. 2018). We have also examined the environmental factors that induce cassava flowering under field conditions in Asian regions (Tokunaga et al. 2020). We believe that if flower-promotion technology can be developed and utilized by cassava research organizations around the world, great advances can be rapidly made in the molecular breeding of cassava.

Genetic transformation and gene editing technologies offer great potential to augment breeding by integrating new traits into cassava germplasm for breeders to utilize, and in some cases to circumvent the need for lengthy breeding programs by introducing beneficial traits into existing farmer-preferred varieties. Transgenesis and gene editing in cassava relies mostly on *Agrobacterium*-mediated transformation using friable embryogenic callus (FEC) as the target tissue for transgene integration followed by recovery of transgenic and gene edited plants (Taylor et al. 1996; Bull et al. 2009; Utsumi et al. 2017). Successful transformation and regeneration of African farmer- and industry-preferred varieties such as TME3, TME7, TME204, and T200, Ebwanatereka, Kibandameno and Serere have been reported (Vanderschuren et al. 2012; Zainuddin et al. 2012; Chetty et al. 2013; Nyaboga et al. 2013; Chauhan et al. 2015). The establishment of an efficient transformation system for Asian varieties, has lagged behind this progress. Recent success in recovery of transgenic plants of the single most important Asian variety KU50 represents major step forward. KU50 is grown on more than 1 million hectares in Thailand and Vietnam and is also cultivated in Indonesia, Cambodia, Myanmar, and the Philippines (Malik et al. 2020). Abilities to genetically modify this variety opens the door for transgenic and gene editing approaches for generating CMD resistant versions of KU50.

Genome-editing technology is a relatively new technology and a powerful tool for crop improvement, shortening the time needed for development of improved varieties (Li 2013; Nekrasov et al. 2013; Shan 2013). Genome editing has been successfully employed in cassava (Odipio et al. 2017; Hummel et al. 2018; Seki et al. 2018) but still faces technical challenges. Firstly, it is necessary to identify useful genes as editing targets for creating desirable traits; including CMD2-mediated resistance. The recent advances in genomics and transformation technologies promises to provide targets for application of genome editing for resistance to CMD in Asian varieties such as KU50.

The development and use of genetically-modified plants can raise public concerns about potential environmental impact and health risks. Alternative methods, such as virus-based vectors and the in vitro-assembled Cas9-gRNA-ribonucleoproteins system, have been utilized in other plant species (Montecillo et al. 2020). The advantage of these approaches is that they do not integrate foreign DNA into the plant genome and reduce off-target mutations, thus avoiding unintended phenotypic traits. A future goal is to develop a transgene-free-editing system that can be used for many different cassava varieties. This can be subsequently used to incorporate SLCMV-resistance and/or other important agricultural traits into superior Asian cultivars of cassava that are already accepted by local farmers and starch factories.

## Conclusion and future perspective

Cassava is an important food source for carbohydrates in southeast Asia and serves as a cash crop for food and industrial materials. The demand for cassava production will continue to increase as economic development continues in Southeast Asia. CMD is now established in Southeast Asia and is spreading. A recent survey in Vietnam indicated that a 16–33% decrease in yield occurred when SLCMV-infected cuttings were used for planting (Supplementary Table 1). This would result in serious economic losses if the CMD epidemic was to become widespread throughout the region.

The SATREPS project developed pest-management tools and a healthy-seedling-production system that can be used to avoid the yield loss of cassava caused by pests and disease such as CMD. CIAT and IITA have introduced CMD-resistant and other useful materials into Vietnam. To provide a genetic solution to the problems that cassava producers face in this region, SATREPS has developed new techniques and methodologies for molecular breeding. The strategies discussed in the present review can be used to address the challenge in Southeast Asia to generate new cassava varieties with high resistance to CMD, as well as other desirable traits. It is hoped that this project and our collaborative efforts will help to strengthen the technical and institutional capacities that are needed to support sustainable development and management systems which will make a significant contribution to food availability, employment, and greater economic opportunities in Southeast Asia.

## Supplementary Information

Below is the link to the electronic supplementary material.

Supplementary file1 (XLSX 16 kb)

Supplementary file2 (DOCX 164 kb)
