# Supplementary material for: Cassava mosaic disease and its management in Southeast Asia
Source: Plant Mol Biol. 2021 Jul 9;109(3):301–11. doi: 10.1007/s11103-021-01168-2 (PMC9162994; doi:10.1007/s11103-021-01168-2)
Supplement: Supplementary file 2 — Supplementary file2 (DOCX 164 kb) [file 11103_2021_1168_MOESM2_ESM.docx]

**Supplementary Fig 1. CMD resistance of C33.**

(A) Typical appearance of C33 and KM419 grown for 4 months in an CMD-infected field in Tay Ninh province, Vietnam. (B) The percentage of CMD-symptomatic plants from a survey conducted from October 2018 to September 2019. A similar result was obtained in 2020. The highest CMD score for each plant was recorded at 1, 3, 6, 9 months after planting. A total of 60 plants of each variety were monitored to determine the percentage of CMD symptomatic plants.
